# Crystal structure and Hirshfeld surface analysis of 1-(2-amino-4-methyl-1,3-thia­zol-5-yl)ethan-1-one

**DOI:** 10.1107/S2056989023007181

**Published:** 2023-09-08

**Authors:** Elnur Z. Huseynov, Mehmet Akkurt, Ivan Brito, Ajaya Bhattarai, Rovnag M. Rzayev, Khammed A. Asadov, Abel M. Maharramov

**Affiliations:** a Department of Chemistry, Baku State University, Z. Khalilov str. 23, Az, 1148 Baku, Azerbaijan; b Department of Physics, Faculty of Sciences, Erciyes University, 38039 Kayseri, Türkiye; c Departamento de Química, Facultad de Ciencias Básicas, Universidad de Antofagasta, Avenida Angamos 601, Casilla 170, Antofagasta 1240000, Chile; d Department of Chemistry, M.M.A.M.C. (Tribhuvan University) Biratnagar, Nepal; e "Composite Materials" Scientific Research Center, Azerbaijan State Economic University (UNEC), H. Aliyev str. 135, Az1063, Baku, Azerbaijan; Katholieke Universiteit Leuven, Belgium

**Keywords:** crystal structure, thia­zole derivatives, hydrogen bonds, dimers, Hirshfeld surface analysis

## Abstract

In the title compound, C_6_H_8_N_2_OS, all atoms except for the methyl H atoms are coplanar, with a maximum deviation of 0.026 (4) Å. In the crystal, pairs of mol­ecules are linked by N—H⋯N hydrogen bonds, forming *R* ^2^_2_(8) ring motifs. Dimers are connected by N—H⋯O hydrogen bonds, forming layers parallel to the (102) plane. Consolidating the mol­ecular packing, these layers are connected by C—H⋯π inter­actions between the center of the 1,3-thia­zole ring and the H atom of the methyl group attached to it, as well as C=O⋯π inter­actions between the center of the 1,3-thia­zole ring and the O atom of the carboxyl group. According to a Hirshfeld surface study, H⋯H (37.6%), O⋯H/H⋯O (16.8%), S⋯H/H⋯S (15.4%), N⋯H/H⋯N (13.0%) and C⋯H/H⋯C (7.6%) inter­actions are the most significant contributors to the crystal packing.

## Abstract 2

In the crystal, pairs of mol­ecules are linked by N—H⋯N hydrogen bonds, forming 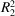 (8) ring motifs. Dimers are connected by N—H⋯O hydrogen bonds, forming layers parallel to the (102) plane. These layers are connected by C—H⋯π and C=O⋯π inter­actions, consolidating the mol­ecular packing.

## 1. Chemical context

**Figure 1:**
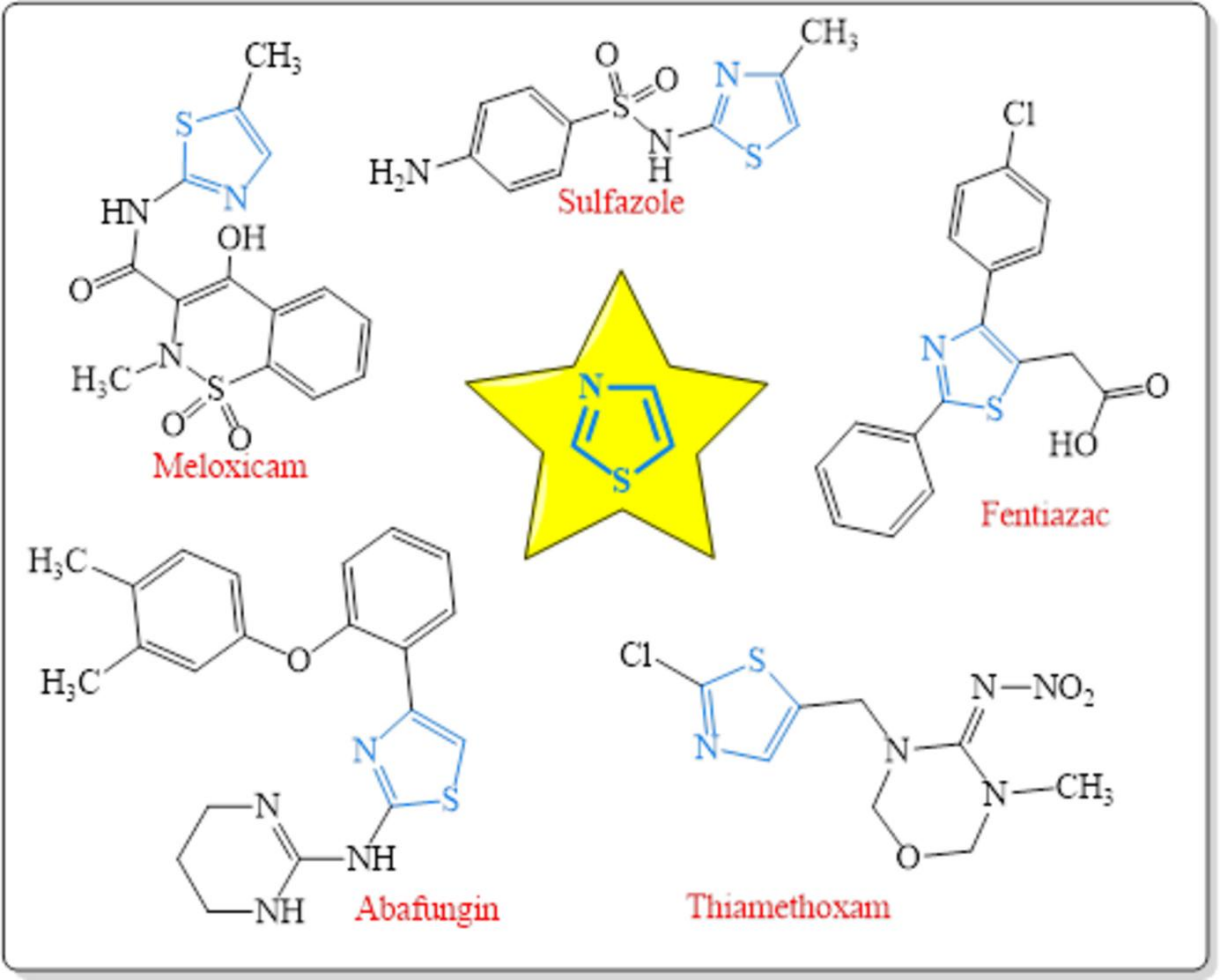
Some marketed drugs containing the thia­zole moiety.

Heterocyclic aromatic systems are the most important and manifold compounds in organic chemistry (Maharramov *et al.*, 2011*b* ▸; Abdelhamid *et al.*, 2014 ▸). Organic synthesis is developing intensely with newer aromatic heterocyclic compounds that are obtained for diverse medicinal and commercial purposes (Khalilov *et al.*, 2021 ▸). Nowadays, applications of five- and six-membered ring heterocycles have expanded in different branches of chemistry, including sustainable chemistry (Montes *et al.*, 2018 ▸), drug design and development (Tas *et al.*, 2023 ▸) and materials sciences (Yin *et al.*, 2020 ▸). The thia­zole core is the most common five-membered heteroaromatic ring system in azole heterocycles (Yadigarov *et al.*, 2009 ▸; Khalilov, 2021 ▸). Thia­zoles have potent medicinal applications as it is an essential core scaffold present in many natural (thi­amine, penicillin) and synthetic medicinally important compounds (Chhabria *et al.*, 2016 ▸) such as sulfazole, ritonavir, abafungin, fanetizole, meloxicam, fenti­azac, nizatidine, thia­methoxam, *etc*. (Fig. 1 ▸). On the other hand, there have been a variety of significant examples of thia­zole derivatives used as target products as well as synthetic inter­mediates (Akkurt *et al.*, 2018 ▸; Kekeçmuhammed *et al.*, 2022 ▸). 
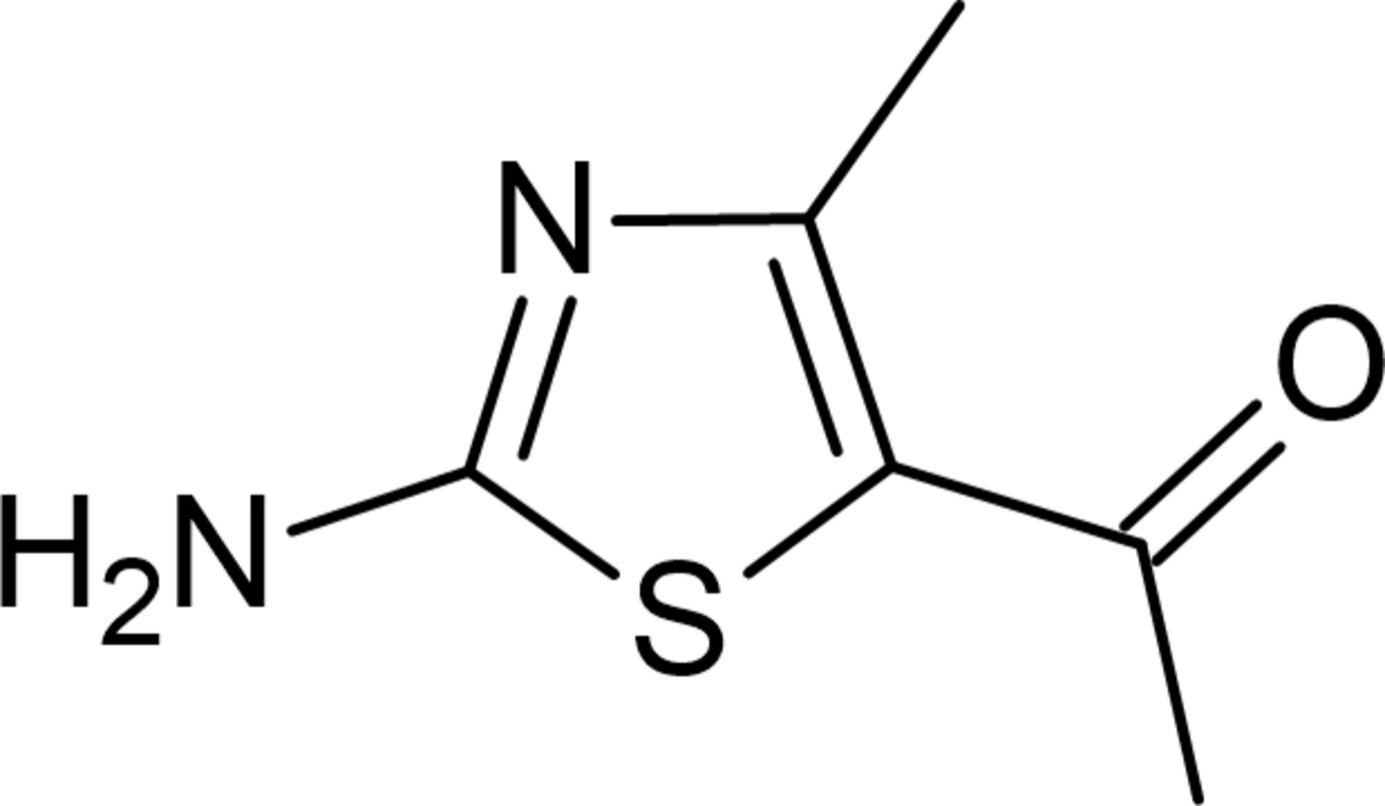


In a continuation of our investigations of heterocyclic systems with biological activity and in the framework of ongoing structural studies (Maharramov *et al.*, 2011*a* ▸; Askerov *et al.*, 2020 ▸; Karimli *et al.*, 2023 ▸), we report here the crystal structure and Hirshfeld surface analysis of the title compound, 1-(2-amino-4-methyl-1,3-thia­zol-5-yl)ethan-1-one.

## 2. Structural commentary

**Figure 2:**
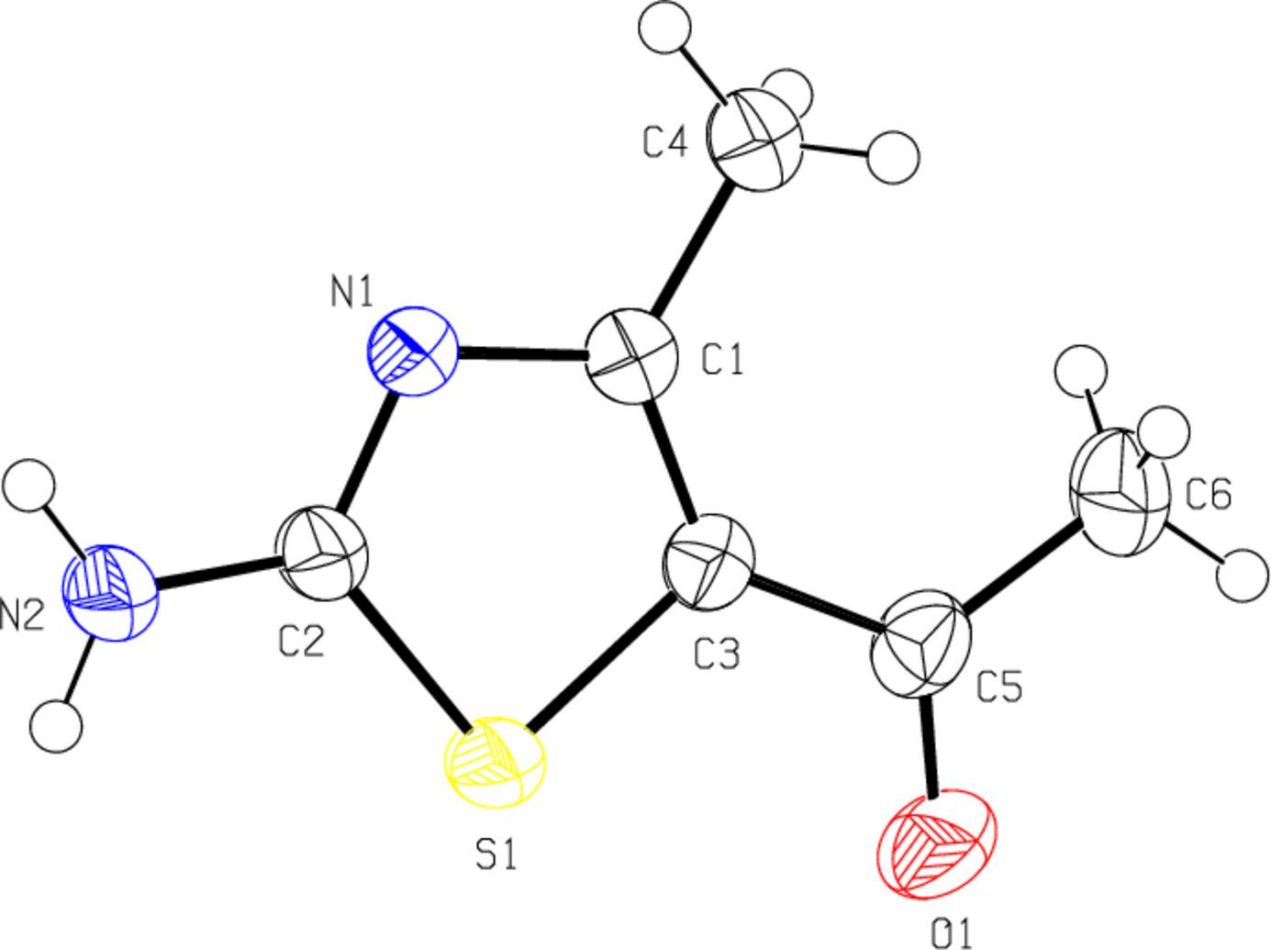
The mol­ecular structure of the title compound, showing the atom labeling and displacement ellipsoids drawn at the 50% probability level.

In the title compound, Fig. 2 ▸, all atoms except for the methyl H atoms are coplanar, with a maximum deviation of 0.026 (4) Å for C6. The geometric parameters of the title compound are normal and comparable to those of related compounds listed in the *Database survey* section.

## 3. Supra­molecular features and Hirshfeld surface analysis

**Figure 3:**
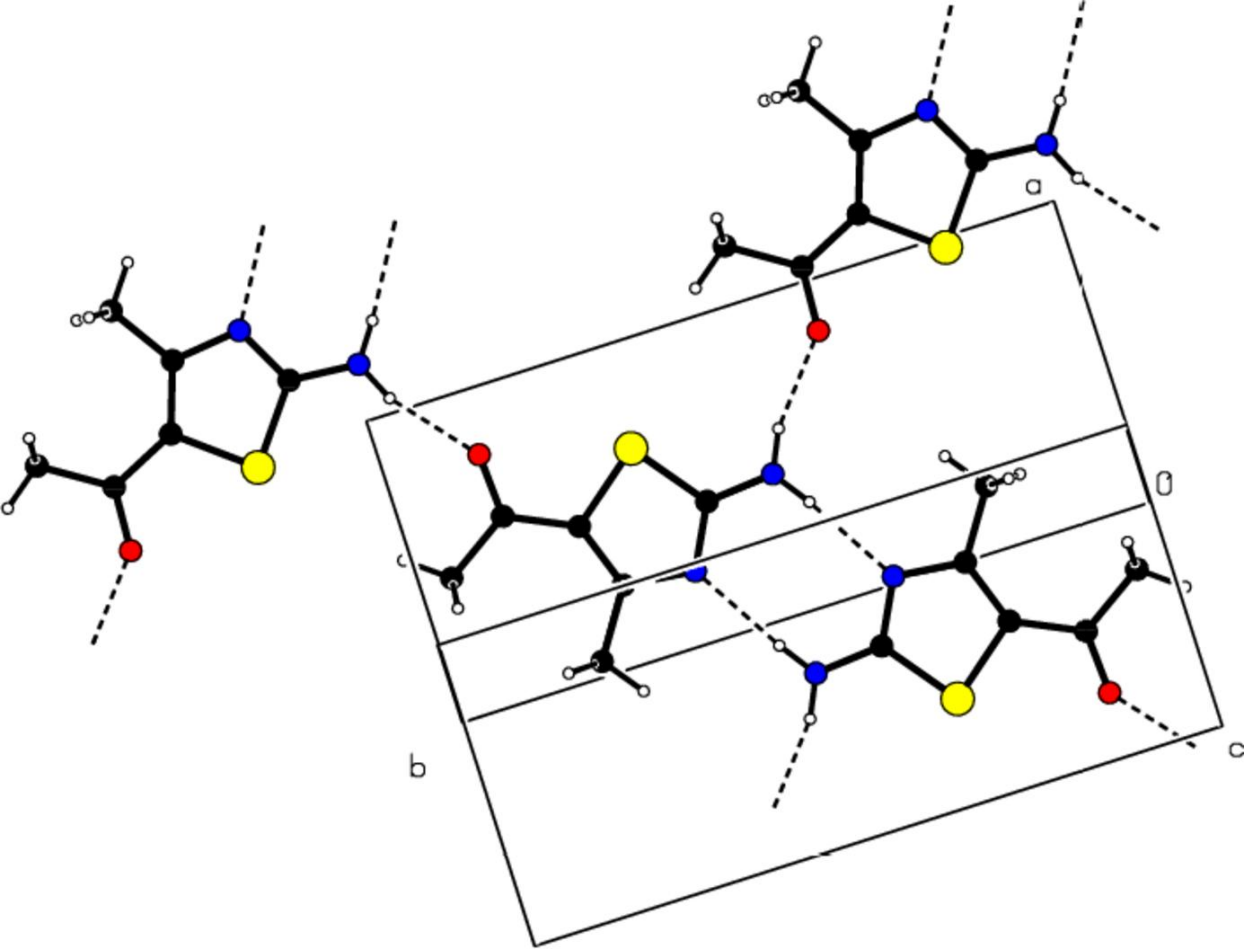
Partial view of the N—H⋯N and N—H⋯O bonds in the (102) plane of the title compound.

**Figure 4:**
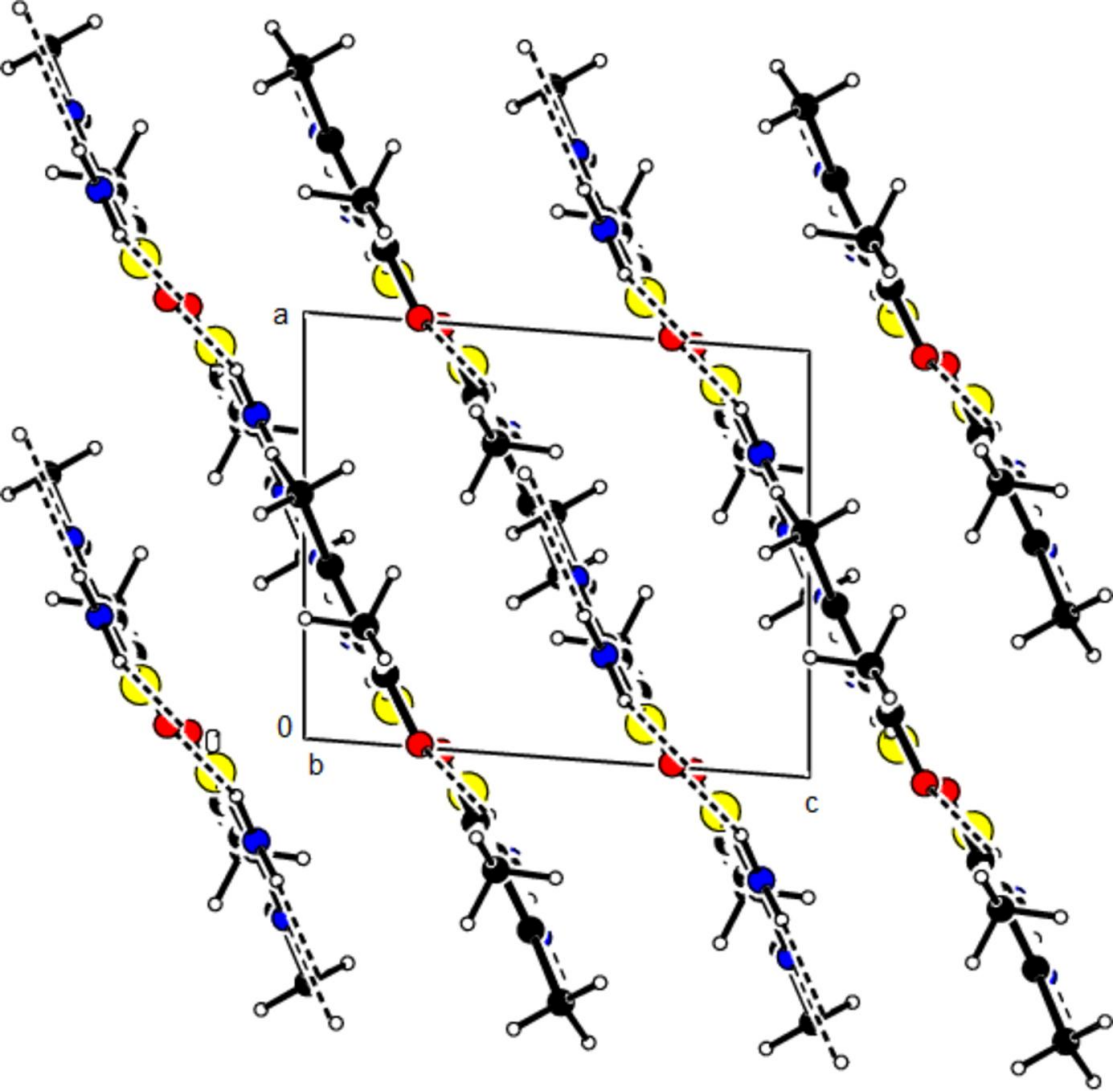
View of the packing of the title compound along the *b*-axis.

**Figure 5:**
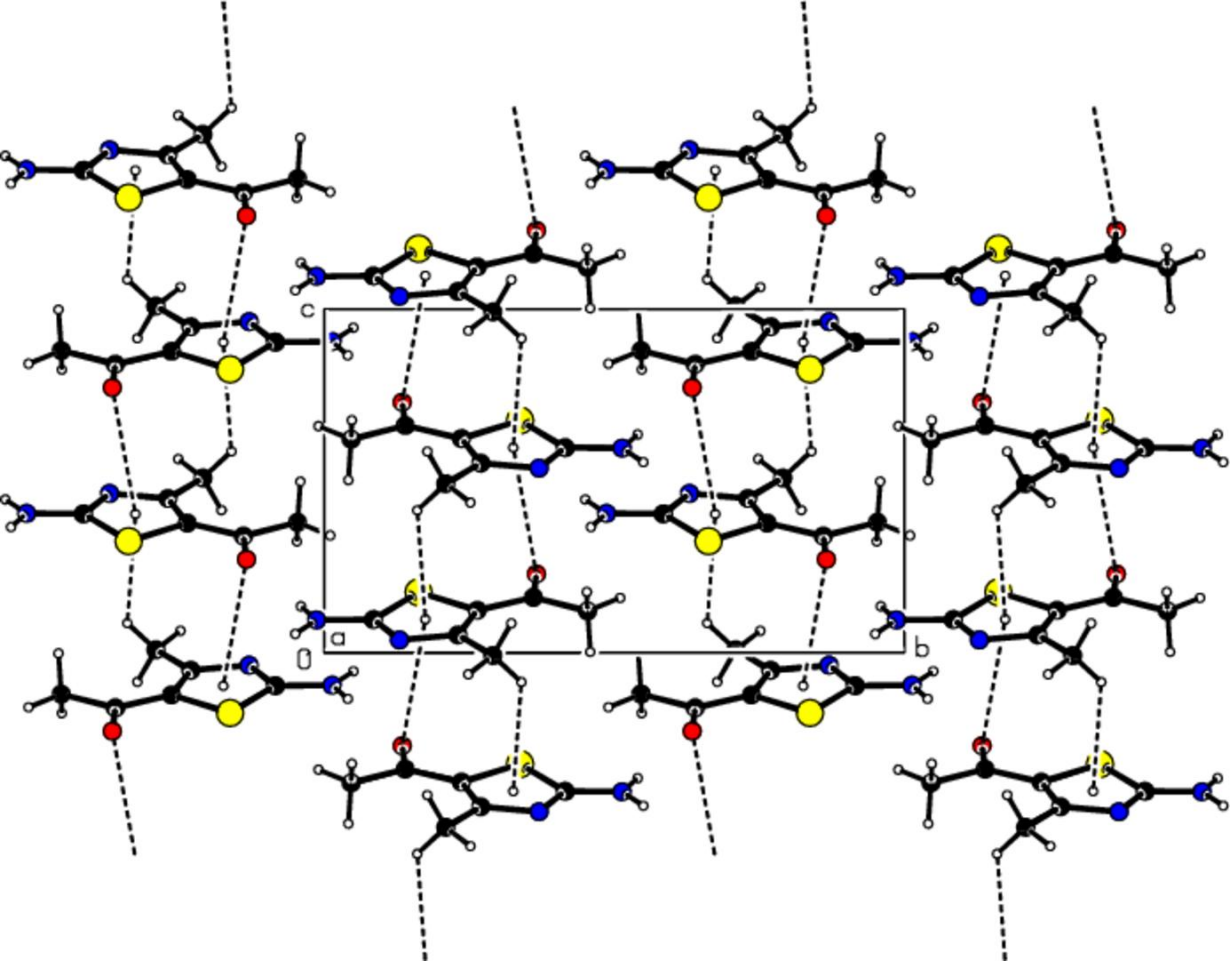
View of the C—H⋯π and C=O⋯π inter­actions of the title compound down the *a* axis.

In the crystal, pairs of mol­ecules are linked by N—H⋯N hydrogen bonds, forming 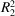 (8) ring motifs (Bernstein *et al.*, 1995 ▸; Table 1 ▸, Fig. 3 ▸). Dimers are connected by N—H⋯O hydrogen bonds, forming layers parallel to the (102) plane (Table 1 ▸, Fig. 4 ▸). Consolidating the mol­ecular packing, these layers are connected by C—H⋯π inter­actions between the center of the 1,3-thia­zole ring and the H atom of the methyl group attached to it, as well as C=O⋯π inter­actions between the center of the 1,3-thia­zole ring and the O atom of the carboxyl group (Table 1 ▸, Figs. 5 ▸ and 6 ▸).

**Figure 7:**
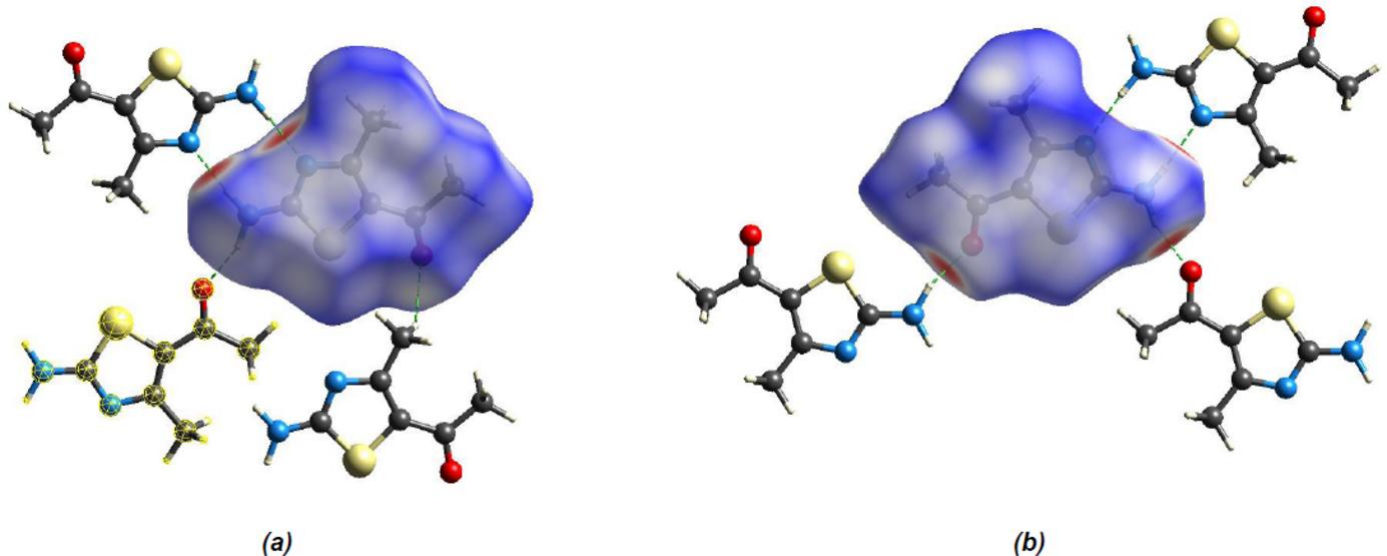
(*a*) Front and (*b*) back sides of the three-dimensional Hirshfeld surface of the title compound mapped over *d*_norm_, with a fixed color scale of −0.5624 to 0.9850 a.u.

**Figure 8:**
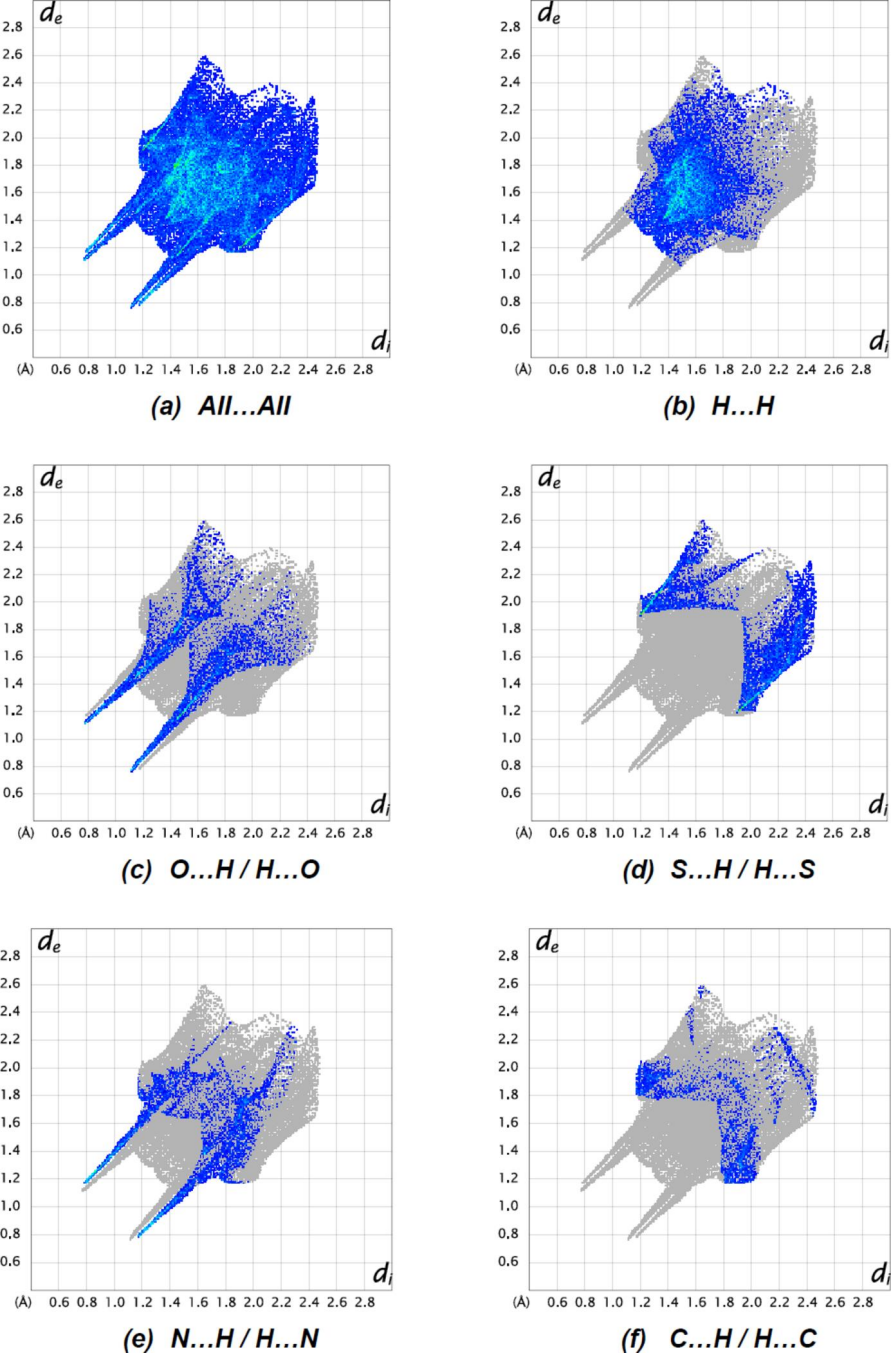
The two-dimensional fingerprint plots of the title compound, showing (*a*) all inter­actions, and delineated into (*b*) H⋯H, (*c*) O⋯H/H⋯O, (*d*) S⋯H/H⋯S, (*e*) N⋯H/H⋯N and (*f*) C⋯H/H⋯C inter­actions. [*d*_e_ and *d*_i_ represent the distances from a point on the Hirshfeld surface to the nearest atoms outside (external) and inside (inter­nal) the surface, respectively].

*Crystal Explorer 17.5* (Spackman *et al.*, 2021 ▸) was used to generate Hirshfeld surfaces and two-dimensional fingerprint plots in order to qu­antify the inter­molecular inter­actions in the crystal. The Hirshfeld surfaces were mapped over *d*_norm_ in the range −0.5624 (red) to 0.9850 (blue) a.u. (Fig. 7 ▸). The inter­actions given in Table 2 ▸ play a key role in the mol­ecular packing of the title compound. The most important inter­atomic contact is H⋯H as it makes the highest contribution to the crystal packing (37.6%, Fig. 8 ▸ *b*). Other major contributors are O⋯H/H⋯O (16.8%, Fig. 8 ▸ *c*), S⋯H/H⋯S (15.4%, Fig. 8 ▸ *d*), N⋯H/H⋯N (13.0%, Fig. 8 ▸ *e*) and C⋯H/H⋯C (7.6%, Fig. 8 ▸ *f*) inter­actions. Other, smaller contributions are made by S⋯C/C⋯S (2.7%), C⋯O/O⋯C (2.6%), C⋯C (1.8%), N⋯C/C⋯N (1.5%), S⋯O/O⋯S (0.8%), S⋯N/N⋯S (0.1%) and O⋯N/N⋯O (0.1%) inter­actions.

## 4. Database survey

A search of the Cambridge Structural Database (CSD, Version 5.43, last update November 2022; Groom *et al.*, 2016 ▸) for the central five-membered ring *1,3-thia­zole* yielded five compounds related to the title compound, *viz*. CSD refcodes IXAMAV (Kennedy *et al.*, 2004*a* ▸), ABEGAQ (Kennedy *et al.*, 2004*b* ▸), FEFKUY (Hazra *et al.*, 2012 ▸), DUTZEY (Chen & Xu, 2010 ▸) and LAMQOJ (Fait *et al.*, 2021 ▸).

In the crystal of IXAMAV, the supra­molecular network is based upon N—H⋯N hydrogen-bonded centrosymmetric dimers linked by N—H⋯O contacts. ABEGAQ forms a supra­molecular network based on N—H⋯N hydrogen-bonded centrosymmetric dimers that are linked in turn by N—H⋯O contacts. In the crystal of FEFKUY, an inter­play of O—H⋯N and C—H⋯O hydrogen bonds connects the mol­ecules to form *C*(6) 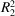 (8) polymeric chains, which are further linked *via* weak C—H⋯O hydrogen bonds into a two-dimensional supra­molecular framework. The crystal structure of DUTZEY involves inter­molecular N—H⋯N hydrogen bonds. In the crystal of LAMQOJ, weak C—H⋯N hydrogen bonds build up a wavy layer of mol­ecules in the (011) plane. The layers are stacked in the [100] direction by weak π–π stacking inter­actions between the 1,3-thia­zole rings.

## 5. Synthesis and crystallization

The title compound was synthesized using a reported procedure (Donald *et al.*, 2012 ▸), and colorless crystals were obtained upon recrystallization from an ethanol/water (3:1) solution at room temperature.

## 6. Refinement

Crystal data, data collection and structure refinement details are summarized in Table 3 ▸. All H atoms were placed in calculated positions (C—H = 0.96 Å and N—H = 0.86 Å) and refined as riding with *U*_iso_(H) = 1.2*U*_eq_(N) for the NH_2_ group and 1.5*U*_eq_(C) for CH_3_ groups.

## Supplementary Material

Crystal structure: contains datablock(s) I. DOI: 10.1107/S2056989023007181/vm2288sup1.cif

Structure factors: contains datablock(s) I. DOI: 10.1107/S2056989023007181/vm2288Isup2.hkl

Supporting information file. DOI: 10.1107/S2056989023007181/vm2288Isup3.cml

CCDC reference: 2288949

Additional supporting information:  crystallographic information; 3D view; checkCIF report

## Figures and Tables

**Figure 6:**
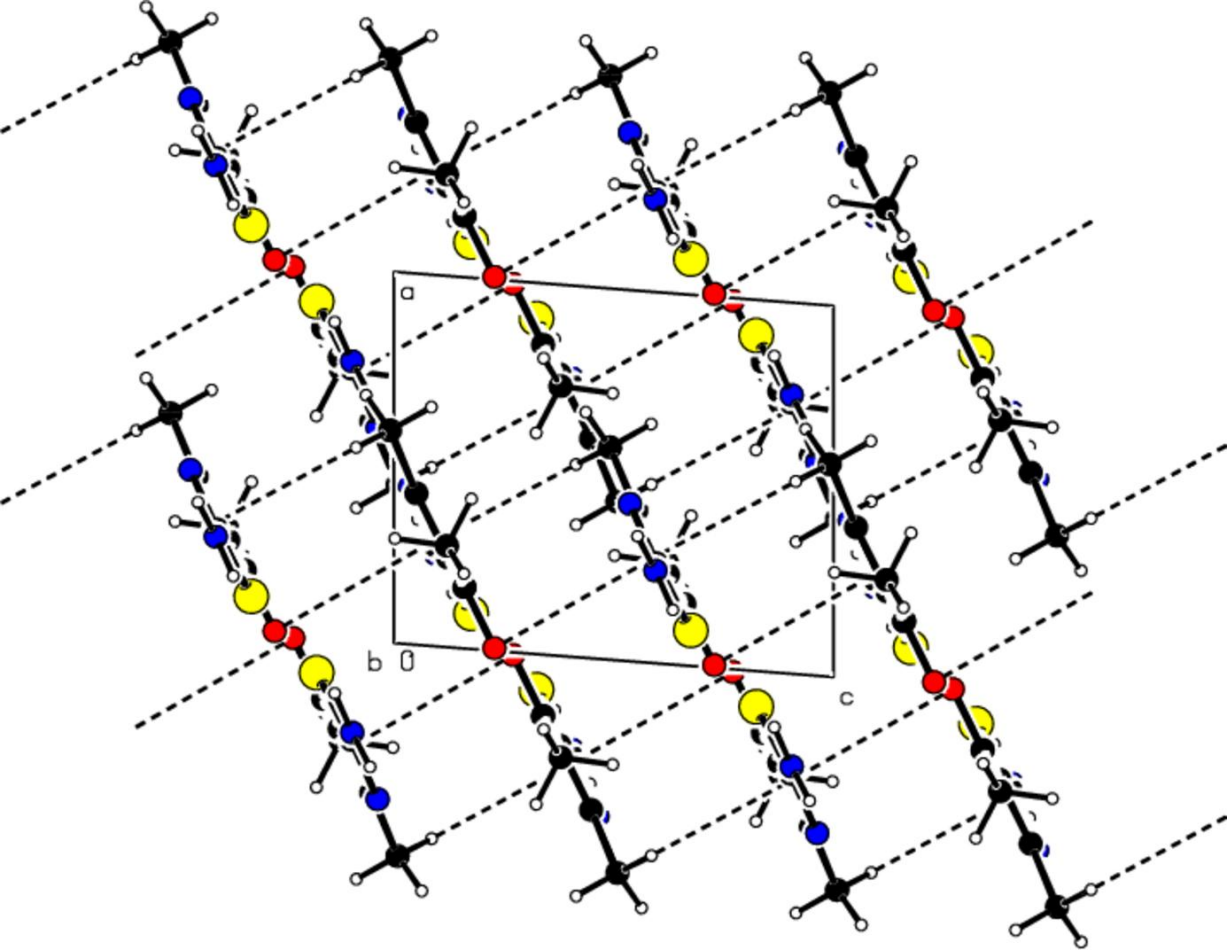
View of the C—H⋯π and C=O⋯π inter­actions of the title compound down the *b* axis.

**Table 1:** Hydrogen-bond geometry (Å, °) *Cg*1 is the centroid of the (N1/S1/C1–C3) 1,3-thia­zole ring.

*D*—H⋯*A*	*D*—H	H⋯*A*	*D*⋯*A*	*D*—H⋯*A*
N2—H1*A*⋯N1^i^	0.86	2.11	2.963 (4)	175
N2—H1*B*⋯O1^ii^	0.86	2.02	2.835 (4)	158
C4—H4*B*⋯*Cg*1^iii^	0.96	2.89	3.603 (4)	132

Symmetry codes: (i) 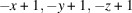 ; (ii) 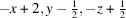 ; (iii) 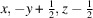 .

**Table 2:** Summary of short inter­atomic contacts (Å) in the title compound

O1⋯H4*A*	2.69	1 + *x*, *y*, *z*
O1⋯H1*B*	2.02	2 − *x*, 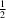 + *y*, 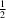 − *z*
C1⋯H4*B*	3.09	*x*, 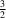 − *y*, − 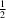 + *z*
H1*A*⋯N1	2.11	1 − *x*, 1 − *y*, 1 − *z*
N2⋯H6*B*	2.89	1 − *x*, − 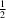 + *y*, 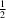 − *z*

**Table 3:** Experimental details

Crystal data	
Chemical formula	C_6_H_8_N_2_OS
*M*_r_	156.20
Crystal system, space group	Monoclinic, *P*2_1_/*c*
Temperature (K)	296
*a*, *b*, *c* (Å)	6.7445 (15), 13.498 (3), 8.010 (2)
β (°)	94.421 (7)
*V* (Å^3^)	727.1 (3)
*Z*	4
Radiation type	Mo *K*α
μ (mm^−1^)	0.37
Crystal size (mm)	0.60 × 0.45 × 0.35

Data collection	
Diffractometer	Bruker APEXII CCD
Absorption correction	Multi-scan (*SADABS*; Krause *et al.*, 2015 ▸
*T*_min_, *T*_max_	0.649, 0.745
No. of measured, independent and observed [*I* > 2σ(*I*)] reflections	14701, 1492, 940
*R*_int_	0.144
(sin θ/λ)_max_ (Å^−1^)	0.626

Refinement	
*R*[*F* ^2^ > 2σ(*F* ^2^)], *wR*(*F* ^2^), *S*	0.050, 0.142, 1.04
No. of reflections	1492
No. of parameters	93
H-atom treatment	H-atom parameters constrained
Δρ_max_, Δρ_min_ (e Å^−3^)	0.29, −0.28

Computer programs: *APEX2* and *SAINT* (Bruker, 2016 ▸), *SHELXT2018/2* (Sheldrick, 2015*a* ▸), *SHELXL2018/3* (Sheldrick, 2015*b* ▸), *ORTEP-3 for Windows* (Farrugia, 2012 ▸) and *PLATON* (Spek, 2020 ▸).

## supplementary crystallographic information

### Crystal data

**Table:**

C_6_H_8_N_2_OS	*F*(000) = 328
*M**_r_* = 156.20	*D*_x_ = 1.427 Mg m^−^^3^
Monoclinic, *P*2_1_/*c*	Mo *K*α radiation, λ = 0.71073 Å
*a* = 6.7445 (15) Å	Cell parameters from 2051 reflections
*b* = 13.498 (3) Å	θ = 3.0–26.4°
*c* = 8.010 (2) Å	µ = 0.37 mm^−^^1^
β = 94.421 (7)°	*T* = 296 K
*V* = 727.1 (3) Å^3^	Prism, colourless
*Z* = 4	0.60 × 0.45 × 0.35 mm

### Data collection

**Table:**

Bruker APEXII CCD diffractometer	940 reflections with *I* > 2σ(*I*)
φ and ω scans	*R*_int_ = 0.144
Absorption correction: multi-scan (SADABS; Krause *et al.*, 2015	θ_max_ = 26.4°, θ_min_ = 3.0°
*T*_min_ = 0.649, *T*_max_ = 0.745	*h* = −8→8
14701 measured reflections	*k* = −16→16
1492 independent reflections	*l* = −10→10

### Refinement

**Table:**

Refinement on *F*^2^	0 restraints
Least-squares matrix: full	Hydrogen site location: inferred from neighbouring sites
*R*[*F*^2^ > 2σ(*F*^2^)] = 0.050	H-atom parameters constrained
*wR*(*F*^2^) = 0.142	*w* = 1/[σ^2^(*F*_o_^2^) + (0.0616*P*)^2^ + 0.4474*P*] where *P* = (*F*_o_^2^ + 2*F*_c_^2^)/3
*S* = 1.04	(Δ/σ)_max_ < 0.001
1492 reflections	Δρ_max_ = 0.29 e Å^−^^3^
93 parameters	Δρ_min_ = −0.28 e Å^−^^3^

### Special details

**Table:**

Geometry. All esds (except the esd in the dihedral angle between two l.s. planes) are estimated using the full covariance matrix. The cell esds are taken into account individually in the estimation of esds in distances, angles and torsion angles; correlations between esds in cell parameters are only used when they are defined by crystal symmetry. An approximate (isotropic) treatment of cell esds is used for estimating esds involving l.s. planes.

### Fractional atomic coordinates and isotropic or equivalent isotropic displacement parameters (Å^2^)

**Table:**

	*x*	*y*	*z*	*U*_iso_*/*U*_eq_	
C1	0.5920 (5)	0.7303 (2)	0.4486 (4)	0.0354 (8)	
C2	0.7302 (5)	0.5848 (2)	0.4036 (4)	0.0356 (8)	
C3	0.7583 (5)	0.7636 (2)	0.3769 (4)	0.0349 (8)	
C4	0.4274 (5)	0.7905 (3)	0.5084 (5)	0.0446 (9)	
H4A	0.356539	0.822440	0.414759	0.067*	
H4B	0.480953	0.839710	0.585759	0.067*	
H4C	0.338231	0.748209	0.563299	0.067*	
C5	0.8348 (5)	0.8605 (3)	0.3393 (4)	0.0408 (9)	
C6	0.7269 (6)	0.9538 (3)	0.3790 (5)	0.0567 (11)	
H6A	0.718170	0.958584	0.497742	0.085*	
H6B	0.595477	0.952624	0.323674	0.085*	
H6C	0.798079	1.010036	0.340806	0.085*	
N1	0.5761 (4)	0.62959 (19)	0.4631 (4)	0.0359 (7)	
N2	0.7505 (4)	0.4865 (2)	0.4051 (4)	0.0498 (9)	
H1A	0.661573	0.449933	0.445684	0.060*	
H1B	0.852593	0.459698	0.365376	0.060*	
S1	0.90402 (13)	0.66254 (6)	0.32437 (12)	0.0412 (3)	
O1	0.9925 (4)	0.8657 (2)	0.2713 (4)	0.0571 (8)	

### Atomic displacement parameters (Å^2^)

**Table:**

	*U*^11^	*U*^22^	*U*^33^	*U*^12^	*U*^13^	*U*^23^
C1	0.0349 (18)	0.0323 (17)	0.039 (2)	0.0005 (15)	0.0045 (15)	−0.0011 (15)
C2	0.0348 (18)	0.0287 (16)	0.044 (2)	0.0034 (14)	0.0063 (15)	0.0031 (15)
C3	0.0343 (18)	0.0308 (17)	0.040 (2)	−0.0023 (14)	0.0035 (15)	0.0009 (15)
C4	0.042 (2)	0.0320 (18)	0.061 (2)	0.0032 (16)	0.0105 (18)	−0.0005 (17)
C5	0.0410 (19)	0.0351 (19)	0.046 (2)	−0.0044 (15)	0.0016 (17)	0.0066 (15)
C6	0.066 (3)	0.0313 (19)	0.075 (3)	0.0012 (19)	0.015 (2)	0.0054 (19)
N1	0.0318 (14)	0.0294 (14)	0.0476 (18)	−0.0007 (11)	0.0093 (13)	0.0001 (13)
N2	0.0450 (18)	0.0295 (16)	0.078 (2)	0.0006 (13)	0.0263 (17)	0.0004 (15)
S1	0.0371 (5)	0.0341 (5)	0.0547 (6)	0.0002 (4)	0.0172 (4)	0.0035 (4)
O1	0.0474 (16)	0.0460 (16)	0.080 (2)	−0.0083 (12)	0.0182 (15)	0.0148 (14)

### Geometric parameters (Å, º)

**Table:**

C1—N1	1.370 (4)	C4—H4B	0.9600
C1—C3	1.375 (4)	C4—H4C	0.9600
C1—C4	1.484 (5)	C5—O1	1.234 (4)
C2—N1	1.322 (4)	C5—C6	1.501 (5)
C2—N2	1.334 (4)	C6—H6A	0.9600
C2—S1	1.730 (3)	C6—H6B	0.9600
C3—C5	1.446 (5)	C6—H6C	0.9600
C3—S1	1.752 (3)	N2—H1A	0.8600
C4—H4A	0.9600	N2—H1B	0.8600
			
N1—C1—C3	115.6 (3)	O1—C5—C3	118.5 (3)
N1—C1—C4	116.8 (3)	O1—C5—C6	119.6 (3)
C3—C1—C4	127.6 (3)	C3—C5—C6	121.9 (3)
N1—C2—N2	122.3 (3)	C5—C6—H6A	109.5
N1—C2—S1	115.4 (2)	C5—C6—H6B	109.5
N2—C2—S1	122.3 (2)	H6A—C6—H6B	109.5
C1—C3—C5	134.3 (3)	C5—C6—H6C	109.5
C1—C3—S1	109.7 (2)	H6A—C6—H6C	109.5
C5—C3—S1	116.0 (2)	H6B—C6—H6C	109.5
C1—C4—H4A	109.5	C2—N1—C1	110.7 (3)
C1—C4—H4B	109.5	C2—N2—H1A	120.0
H4A—C4—H4B	109.5	C2—N2—H1B	120.0
C1—C4—H4C	109.5	H1A—N2—H1B	120.0
H4A—C4—H4C	109.5	C2—S1—C3	88.60 (15)
H4B—C4—H4C	109.5		
			
N1—C1—C3—C5	−179.3 (4)	N2—C2—N1—C1	179.3 (3)
C4—C1—C3—C5	2.0 (7)	S1—C2—N1—C1	−0.5 (4)
N1—C1—C3—S1	0.0 (4)	C3—C1—N1—C2	0.3 (5)
C4—C1—C3—S1	−178.7 (3)	C4—C1—N1—C2	179.2 (3)
C1—C3—C5—O1	−179.5 (4)	N1—C2—S1—C3	0.4 (3)
S1—C3—C5—O1	1.3 (5)	N2—C2—S1—C3	−179.4 (3)
C1—C3—C5—C6	−0.1 (7)	C1—C3—S1—C2	−0.2 (3)
S1—C3—C5—C6	−179.4 (3)	C5—C3—S1—C2	179.2 (3)

### Hydrogen-bond geometry (Å, º)

*Cg*1 is the centroid of the (N1/S1/C1–C3) 1,3-thiazole ring.

**Table:**

*D*—H···*A*	*D*—H	H···*A*	*D*···*A*	*D*—H···*A*
N2—H1*A*···N1^i^	0.86	2.11	2.963 (4)	175
N2—H1*B*···O1^ii^	0.86	2.02	2.835 (4)	158
C4—H4*B*···*Cg*1^iii^	0.96	2.89	3.603 (4)	132

Symmetry codes: (i) −*x*+1, −*y*+1, −*z*+1; (ii) −*x*+2, *y*−1/2, −*z*+1/2; (iii) *x*, −*y*+1/2, *z*−1/2.
